# Open Treatments for Thoracoabdominal Aortic Aneurysm Repair

**DOI:** 10.14797/mdcvj.1178

**Published:** 2023-03-07

**Authors:** Akiko Tanaka, Holly N. Smith, Hazim J. Safi, Anthony L. Estrera

**Affiliations:** 1 McGovern Medical School at UTHealth Houston, Houston, Texas, US

**Keywords:** thoracoabdominal aortic aneurysm, TAAA, open repair

## Abstract

Thoracoabdominal aortic aneurysms (TAAA) represent a unique pathology that is associated with considerable mortality if untreated. While the advent of endovascular technologies has introduced new modalities for consideration, the mainstay of TAAA treatment remains open surgical repair. However, the optimal conduct of open TAAA repair requires careful consideration of patient risk factors and a collaborative team effort to mitigate the risk of perioperative complications. In this chapter, we briefly outline the history of treating TAAA, preoperative preparation and postoperative care, and our operative techniques for treatment.

## History of Open Thoracoabdominal Aortic Aneurysm Repair

Open repair of thoracoabdominal aortic aneurysm (TAAA) began in 1955 with Etheredge and colleagues,^1^ who were the first to report a successful TAAA open repair using a homograft, followed by DeBakey and colleagues a year later.^2^ The use of cardiopulmonary bypass or left heart bypass were not established at the time, and DeBakey and colleagues used a Dacron graft to shunt between the descending thoracic aorta and infrarenal abdominal aorta to minimize visceral and renal ischemia. By the 1970s, Crawford and colleagues established modern techniques to treat TAAA, which included three principles: (1) the inclusion technique to avoid damage to surrounding structures; (2) the reattachment of the renal and visceral arteries into the larger graft; and (3) the reattachment of the intercostal arteries to prevent paraplegia.^3^

The challenges in repairing TAAA are ischemic insults to multiple organs. In the 1960s, multiple approaches for spinal cord protection, as represented by cerebrospinal fluid (CSF) drainage, were introduced.^4^ The benefit of CSF drainage was confirmed by Hollier and colleagues in the late 1980s but fell into obscurity after Crawford and colleagues reported controversial outcomes in 1991.^5,6^ CSF drain was again proven to be beneficial for spinal cord protection in the 2000s.^7^

Before the 1990s, the “clamp-and-sew” technique was the primary surgical approach for treatment. The use of distal aortic perfusion (DAP)—or partial bypass—was first reported in 1956 by DeBakey and associates and revisited by Connolly and colleagues in 1971.^2,8^ The benefits of DAP include prevention of distal ischemia and reduction of cardiac afterload. It was also confirmed to reduce paraplegia in studies in the 1980s and 1990s.^9,10^ Use of DAP and CSF drainage combined with moderate passive hypothermia has reduced the overall spinal cord ischemia rate after extent I TAAA from 15% to less than 2%, and after extent II TAAA from 33% (50% with clamp time exceeding 40 minutes in the “clamp-and-go” era) to less than 4%.^11^ Although it remains difficult to unequivocally prove the benefit of intercostal reattachment, indirect evidence with improvement of motor evoked potential (MEP) and somatosensory evoked potential (SSEP) responses suggests spinal cord perfusion is improved with potential benefit. This contrasts with those who argue against this adjunct based on the collateral network concept of spinal cord circulation.^12^ Although disputed by some,^12,13^ patent intercostal arteries in the T8-T12 distribution should be reattached at the time of surgery to prevent immediate and delayed paraplegia.^14^ Patients with extent II and III are at risk for spinal cord ischemia, and reimplantation of T8-T12 should be considered regardless of neuromonitoring, if feasible.

The concept of visceral perfusion with a shunt from the descending aorta to both renal arteries, the celiac axis, and the superior mesenteric artery was first described by Korompai and Hayward in 1975.^15^ Later, visceral and renal perfusion techniques using the roller pump became a standard technique to prevent organ ischemia.^10^

## Preoperative Preparation

Preoperative evaluation and preparation of the patient is crucial to optimize the postoperative outcomes after TAAA repair. Patients with severe chronic obstructive pulmonary disease, as suggested by a forced expiratory volume in 1 second of less than 0.8 L/min and a partial pressure of carbon dioxide greater than 45 mm Hg, should be evaluated by a pulmonologist and receive bronchodilators as well as pulmonary rehabilitation before repair. Preoperative renal function is a strong predictor of postoperative mortality after TAAA repair, and glomerular filtration rate is more sensitive than serum creatinine in predicting postoperative outcomes.^16^ Patients with a glomerular filtration rate less than 30 mL/min/1.73m^2^ have a mortality rate of more than 30%. Large aneurysms may cause “aortic dysphagia” as a result of intrinsic compression of the lower esophagus, leading to a nutritionally depleted state that may benefit from preoperative enteral alimentation.

## Classification for Descending Thoracic and Thoracoabdominal Aortic Aneurysm

**Figure 1:**
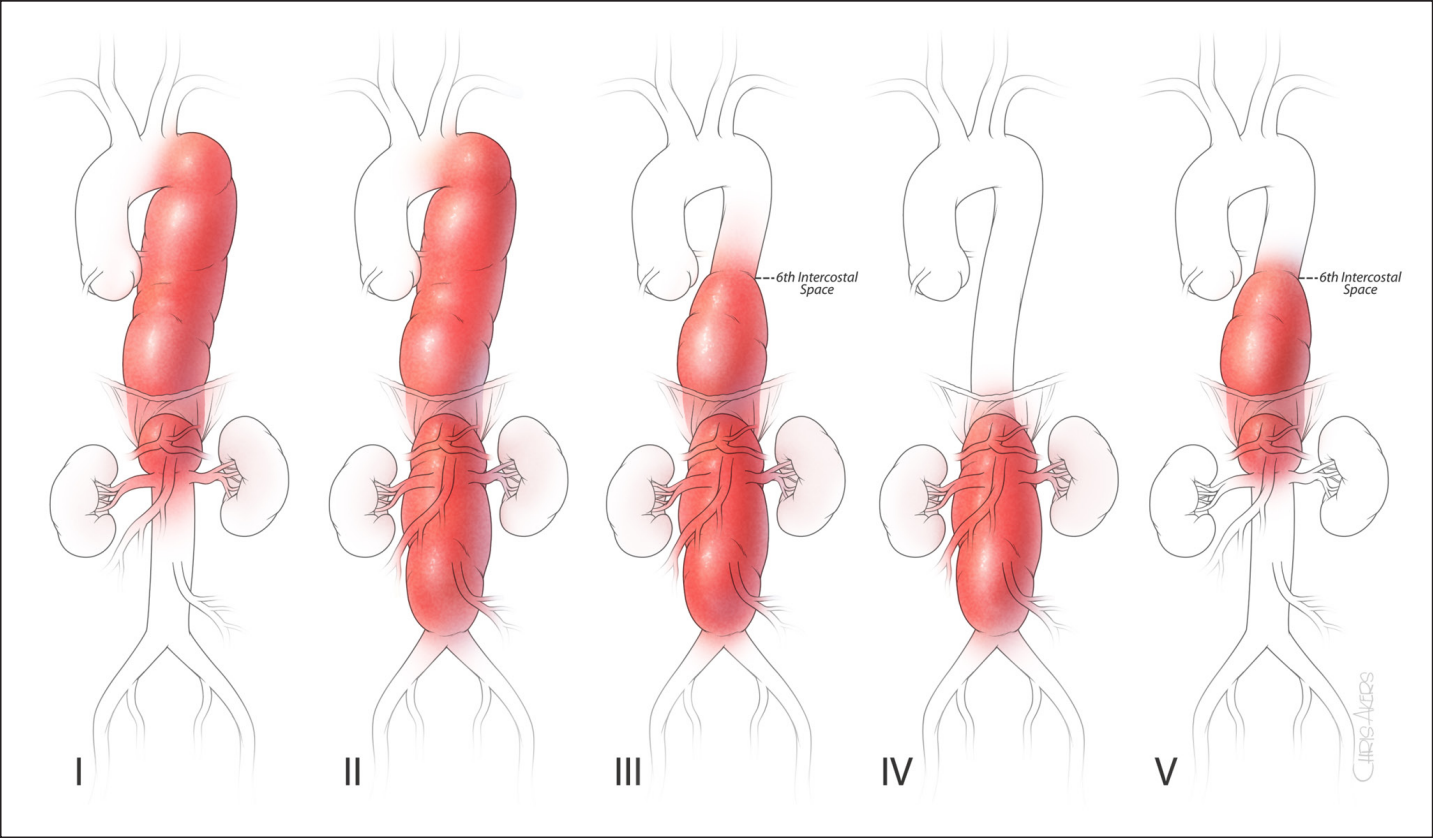
Classifications of thoracoabdominal aortic aneurysms—the scheme of thoracoabdominal aortic aneurysms depicted by extent of aneurysm.

A classification for TAAA describes five anatomic extents: extent I extends from the left subclavian artery to just above the renal arteries; extent II from the left subclavian to the aortic bifurcation; extent III from the sixth intercostal space to the aortic bifurcation; extent IV from the 12th intercostal space to the aortic bifurcation; and extent V from the sixth intercostal space to just above the renal arteries (Figure 1). This classification has been used in the prediction of complications,^17^ especially the risk of spinal cord ischemia, which is highest for extent II TAAA. In our experience, significant disabling complications (composite of operative mortality, paraplegia/paraparesis, stroke, and dialysis) were more frequently seen in the order of extent II, extent III, extent I, extent V, and extent IV.^17^

## Operative Techniques for Open TAAA Repair

### Anesthesia and Monitoring

After induction of general anesthesia, the patient is intubated with a double-lumen endotracheal tube. We place a large-bore, central, and venous catheter for rapid infusion along with a Swan-Ganz catheter for cardiac evaluation, and we monitor nasal and bladder temperatures for core temperature. Renal temperature is obtained later in the operative field using a needle-probe after exposing the retroperitoneal space. Electroencephalogram, somatosensory evoked potential (SSEP), and motor evoked potential (MEP) are also placed at this point. We then turn the patient to the right decubitus position to insert a CSF drainage catheter and advance 5 to 10 cm at the level of L3/L4, L4/L5, or L5/S1. We monitor the CSF pressure throughout the entire procedure and drain, as needed, to maintain the pressure below 10 mm Hg. Modification of anesthetic technique is required to not interfere with MEP and SSEP monitoring. Induction consists of narcotic loading: fentanyl citrate (10-15 μg/kg), midazolam (50 μg/kg), propofol (0.5 mg/kg), and cisatracurium (0.2 mg/kg). Patients are then maintained on a volatile agent (isoflurane) at 0.5 minimal alveolar concentration. Since 2019, induction has consisted of narcotic loading as follows: fentanyl citrate (2-5 μg/kg), midazolam (30-50 μg/kg), propofol (0.5-1 mg/kg), and rocuronium bromide (0.6-1mg/kg). Patients are then maintained on a volatile agent (sevoflurane) at 0.5 minimal alveolar concentration. We do not give any neuromuscular blockers during MEP monitoring. Cisatracurium 0.1 mg/kg may be given for closing the thoracoabdominal spaces or changing double lumen tracheal tube to single endotracheal tube at the conclusion of the surgery.

SSEPs and MEPs are helpful to determine the conduct of operation, especially the timing of the intercostal artery reconstruction.^18^ An abnormal response is defined as a 10% change in latency or 50% change in amplitude from the baseline. Findings of abnormal SSEP or MEP warrant a series of corrective measures to improve spinal cord perfusion, including increasing the proximal mean blood pressure to at least 90 mm Hg and distal aortic pressure to at least 60 mm Hg. CSF pressure may be reduced by gravity drainage and hemoglobin increased by transfusion. Furthermore, additional patent intercostal arteries should be reimplanted, especially those between T8 and L1, as necessary.

### Positioning, Incision, and Exposure

**Figure 2:**
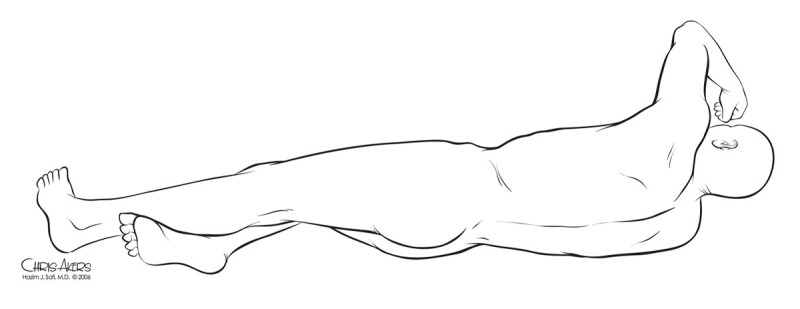
Positioning and skin incision. Note the scapulars are at 90 degrees to the table but hips are rotated for abdominal and femoral exposures.

The patient should be positioned with the scapulars at a right angle to the edge of the operating table, stabilized by a bean bag, with both axillae well padded. The hips are tilted 60 degrees so that both femoral arteries are accessible (Figure 2). The left knee should be flexed and a pillow placed between both lower extremities. The patient is secured to the table with the bean bag vacuumed into a supportive shape. The table should then be flexed and the kidney rest elevated at the flexion point for greater lateral flexion and to improve access to the retroperitoneal space.

For most TAAA procedures, the incision begins at or below the level of the scapula, depending upon the proximal extent, which is then carried down to the costal cartilage along the anterolateral margin of the abdominal wall, lateral to the rectus sheath, to the level of the umbilicus or pubis as dictated by the distal extent of the aneurysm. The incision is extended below the umbilicus for extent II and III, or the incision can end above the umbilicus for extent I and V TAAA repairs. Access to the external iliac arteries may require extension of the incision to the pubis.

**Figure 3:**
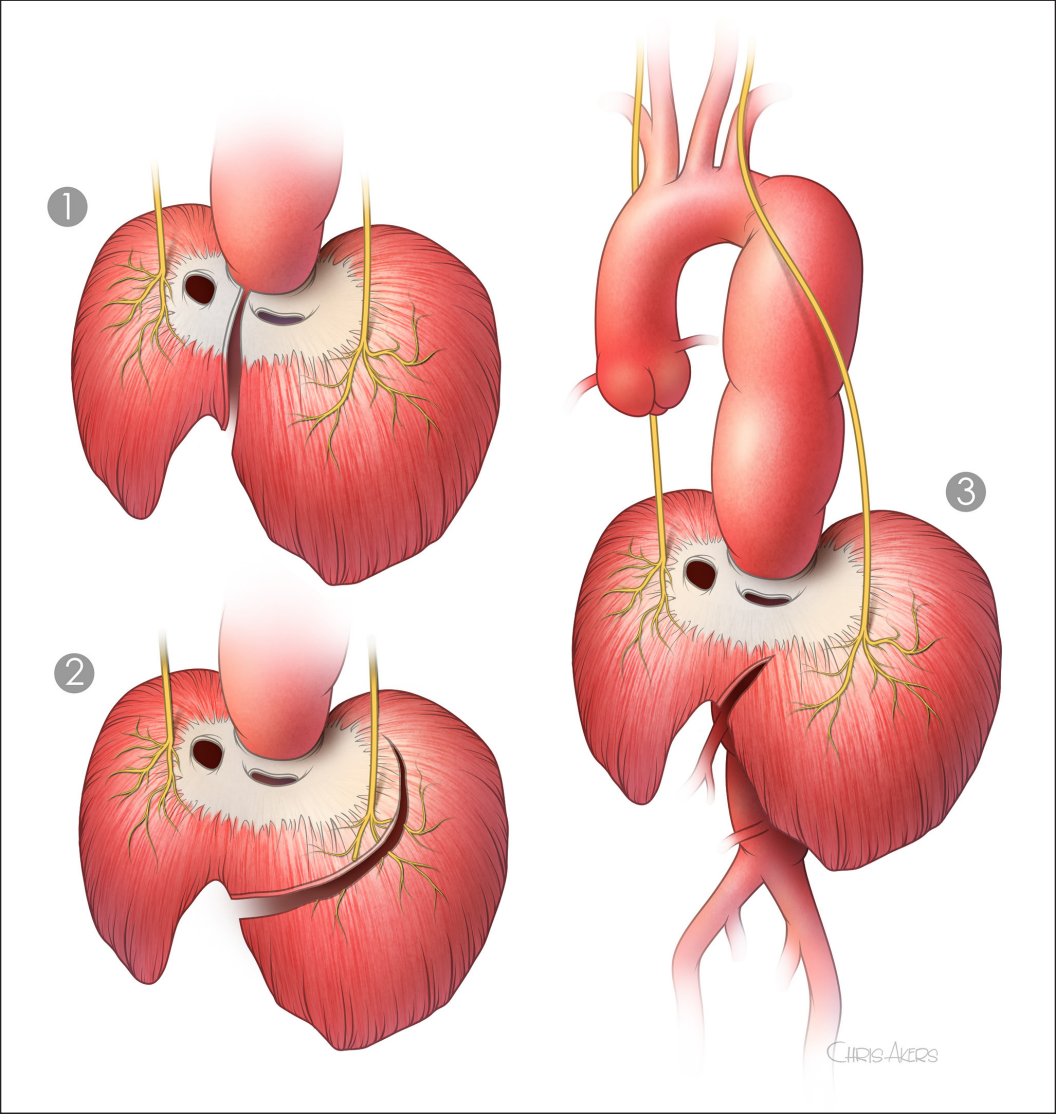
Diaphragm splitting. **(1)** Radial division, **(2)** lateral division, and **(3)** partial lateral division. Partial lateral division of the diaphragm with sparing of the phrenic nerve has been associated with enhanced postoperative recovery of diaphragmatic and pulmonary function. Note that we cut only one-third of the circumference.

The thoracic interspaces are defined by counting down from the first rib. The first rib is slightly difficult to palpate, but the second rib is easily found and confirmed as the posterior scalene muscle attaches to it. We routinely enter the sixth intercostal space for all extents. The chest is entered after the left lung is deflated, and the costal cartilage is cut anteriorly. The ribs above and below may be shingled or divided posteriorly if more proximal exposure is required in the thoracic cavity. The anterior muscular portion of the diaphragm is partially split, with special care to avoid injury to the phrenic nerve and reserve the pulmonary function (Figure 3).^19^

The retroperitoneal space below the diaphragm should be dissected away from the diaphragm prior to the splitting. The left crus of the diaphragm and median arcuate ligament are divided to create an aortic hiatus for the graft.^19^ The retroperitoneal approach is preferred because it prevents the viscera (especially the small bowel) from obscuring the operative field, facilitates closure, and decreases water and heat loss. The left kidney and the viscera are mobilized medially, exposing the aorta from the aortic hiatus to the iliac bifurcation. A large ascending lumbar renal vein is encountered upon exposing the TAAA and must be ligated to avoid bleeding. The initial proximal clamp site just distal to the left subclavian artery can be safely secured with a circumferential blunt dissection using fingers along the lateral border of the trachea.

## Left Heart Bypass

The left inferior pulmonary vein is used to drain the left heart bypass. On exposure of the left inferior pulmonary vein, the pericardium is opened posteriorly to the left phrenic nerve. Upon entering the pericardial cavity, small pericardial effusion is usually encountered. A pledgeted, 3-0 polypropylene purse-string suture is placed in the left inferior pulmonary vein, followed by a transverse incision. The venotomy is dilated, and the cannula is inserted into the left atrium and connected to the centrifugal pump and inline heat exchanger. Although the femoral artery can be directly cannulated using the Seldinger technique when large in caliber, it may be preferable to suture an 8-mm graft onto the femoral artery, as a sleeve for the cannula, to prevent ischemia to the leg. Myoglobinuria that occurs from warm ischemia to the leg with direct femoral artery occlusion for arterial cannulation may contribute to renal dysfunction after TAAA.^20^ Therefore, a sutured graft to the femoral artery may be used to prevent lower-leg ischemia during cannulation. As an alternative to avoid accessing the femoral artery, the distal aortic anastomosis can be performed initially using a premanufactured single-arm branched aortic graft (distal first technique). The sidearm of the tube graft is then connected to the inflow portion of the distal perfusion circuit.

An advantage of using a left heart bypass is the ability to maintain proximal pressure above 100 mm Hg and distal pressure above 60 mm Hg. In addition, the presence of an inline heat exchanger allows the patient to be effectively warmed during the later stages of the procedure.

### Proximal Anastomosis

The proximal clamp is placed either proximally or distally to the left subclavian artery, depending on the presence of atheromatous debris and aortic dissection. Sequential clamping of the distal aorta is used to minimize the ischemic insults to the viscera, kidneys, and spinal cord. Thus, the initial distal aortic clamp is placed at the level of T6, which is then moved distally to the distal descending thoracic aorta and to the infrarenal aorta. After placing the proximal and distal clamps, the proximal descending thoracic aorta is opened and all the thrombus is removed. In patients with chronic dissection, the initial lumen entered is usually a false lumen, and a flap between the false and true lumen must be excised. This step is important to visualize the hidden patent intercostal arteries in a small true lumen. The intercostal arteries within the initial opened segment are all ligated (ie, intercostal arteries above T6). The proximal aorta is circumferentially divided and lifted off the esophagus to prevent an inadvertent esophagograft fistula. Most frequently, 3-0 polypropylene is sufficient to perform proximal anastomosis.

In cases with a fragile aortic wall, such as acute dissection and traumatic injury, 4-0 polypropylene is preferred. In patients with a calcified aorta, running 2-0 polypropylene may be used. The suture line begins at the posterior wall with an anchor technique. The posterior wall is selectively reinforced using a pledgeted suture of 3-0 or 4-0 polypropylene. A felt strip is usually avoided as it makes it difficult to search for the actual bleeding site after the anastomosis. Once that is completed, the anterior anastomosis is continued. This anastomosis is checked for hemostasis by releasing the clamp and occluding the graft. Any bleeding portions of the anastomosis are reinforced using interrupted pledgeted sutures. In cases of young patients, proximal aortic disease, or dissection in the aortic arch, the reversed elephant trunk technique is used to prepare for possible future proximal intervention.

In cases of a previous stent graft, if it has type I endoleak, the graft should be removed and proximal/distal anastomosis should be performed to the native aorta. When the stent graft is well incorporated to the native aorta without signs of type I endoleak, the anastomosis can be performed by suturing the graft to the aortic wall and stent graft. The aorta should be transected at the level of anastomosis to allow for secure anastomosis. A 2-0 polypropylene suture is usually needed because the layers become thick and strong needles are required.

### Distal Anastomosis, Intercostal Artery Reattachment, and Visceral/Renal Artery Reconstruction

**Figure 4:**
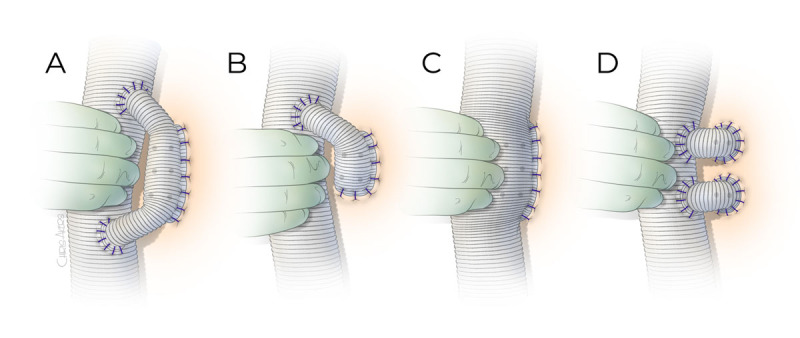
Intercostal artery reattachment techniques. **(A)** A 14-mm Dacron graft is anastomosed to the anterolateral surface of the main body graft as a loop to reconstruct the intercostal arteries in side-to-end fashion. **(B)** Similar to the loop graft but the proximal end may not be anastomosed back to the main body graft if reconstructing less than three pairs of intercostal arteries. The anastomosis to the intercostal arteries is performed in end-to-end fashion by beveling the 14-mm graft. **(C)** A side hole is made large enough to accommodate the intercostal arteries to be reattached and anastomosed as an island patch in side-to-side fashion. **(D)** A 12-mm ×14-mm Dacron graft is used to reattach each pair of intercostal arteries in an end-to-end fashion.

The proximal clamp is now on the graft just distal to the anastomosis, and the distal clamp is moved to the end of the descending aorta. The remaining thoracic aorta is further opened. The patent T8-T12 intercostal arteries are preserved, but others are ligated upon opening the aorta. 3F balloon-tipped occlusion catheters can be used to temporarily occlude the T8-1T12 intercostal arteries to prevent steal phenomenon. The changes in MEPs and SSEPs will dictate when to reattach the T8-T12 intercostal arteries. There are three methods of intercostal reattachment: (1) loop reconstruction using a 14-mm Dacron graft connected to the main body Dacron graft in a side-to-end and side-to-side fashion to the intercostal artery of half loop for small number of pairs (Figure 4); (2) side-to-side reconstruction by creating a side hole in the main Dacron tube graft and anastomosing to the intercostal arteries as an island; and (3) end-to-end reconstruction using an interposing 12-mm by 14-mm Dacron tube graft between the main Dacron graft and each pair of intercostal arteries. If signal changes are observed early during the clamp period, the important patent intercostal arteries within T8 through T12 are reattached immediately. We began using loop reconstruction as it seems to reduce the risk of graft thrombosis compared with direct end-to-end anastomosis from a side-arm graft. Otherwise, the reattachment can be performed with distal anastomosis and weaning off pump prior to protamine reversal.

Then, the distal clamp is further moved below the infrarenal aorta and the visceral aorta is opened. The orifices of the celiac, superior mesenteric, and the left and right renal arteries are identified from the inside. Balloon-tip perfusion catheters are inserted into each orifice. The celiac axis and superior mesenteric artery are perfused with continuous blood while the renal arteries are infused with cold (4°C) crystalloid with potassium into the renal arteries until the left renal temperature reaches below 20°C (usually a total of 200-300 mL), which is supplemented by a bolus dose to maintain the temperature.

Distal anastomosis is preferred prior to visceral and renal reconstruction to restore the pulsatile flow to the pelvis to improve spinal perfusion. In chronic type A or B dissection, the distal anastomosis is typically created in a fenestrated fashion (double barrel) without attempting to incorporate the flap or septum since it is difficult to discern the true from false lumen.

**Figure 5:**
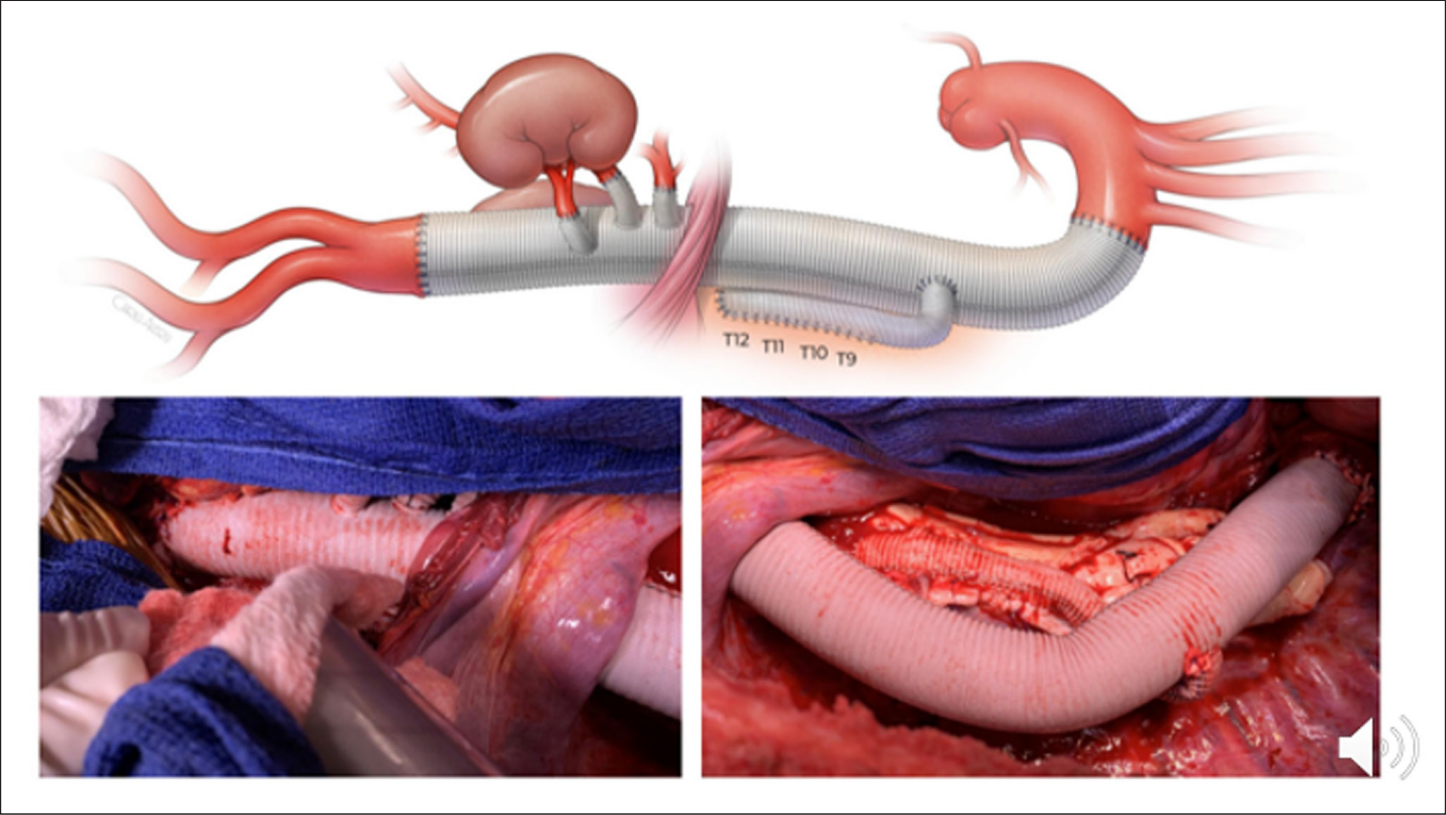
Extent II thoracoabdominal aortic aneurysm repair with side-branched thoracoabdominal aortic graft (STAG).

The distal clamp is released to fill the graft with blood and evacuate the graft of all air and debris by placing the patient in the head-down position. The proximal clamp is slowly released to flush the graft in a prograde manner. The ostia of the visceral and renal vessels are examined and cleaned of atheromatous debris. If the ostia are less than 2 cm apart, then a single-island patch can be created or reattached to the side of the main graft with 3-0 polypropylene suture. If a vessel (most frequently the left renal artery) is separated from the other vessels, then a separate interposition bypass graft is performed using an 8- or 10-mm Dacron tube graft. In cases of Marfan syndrome or other connective tissue disorders in patients younger than 60 years of age, or with ostial separation of greater than 3 cm, a premanufactured side-branched thoracoabdominal aortic graft (STAG) is used with separate bypasses to each of the vessels (Figure 5). This is performed consecutively, starting with the celiac and superior mesenteric arteries and ending with the left renal artery. Creating buttons for each ostium facilitates suturing using running 4-0 polypropylene.

Once the patient is rewarmed to a nasopharyngeal temperature of 36°C and the bladder/rectal temperature is 35°C, DAP is discontinued. All cannulae are removed from the patient and sutures are secured. Heparinization is reversed using protamine sulfate after confirming good pulses in the cannulated femoral artery.

### Hemostasis and Closure

Meticulous attention to hemostasis is mandatory and reduces coagulopathy. It is also important to interrogate the anastomoses. To prevent coagulopathy, hemostasis of muscle edges during thoracoabdominal incision should be managed prior to heparinization. All the mural thrombus in the aorta should be removed to search for intercostal arteries or small branches with patent orifice—and these should be ligated as they may later start to bleed.

When cell salvage amount exceeds 30 to 40 units, appropriate blood products should be given to correct the coagulopathy.^21^ In cases of persistent coagulopathy, vacuum-assisted closure of the abdomen with sponge and pad packing may be instituted. Infusion of platelet-rich plasma may also be considered to reduce the intraoperative bleeding.^22^

Three No. 36 chest tubes are placed: one anteriorly; one posteriorly; and one right-angled above the diaphragm to prevent fluid accumulation in the phrenic sulcus. Before closure, intercostal nerve cryoanalgesia from T4 to T10 are performed to help control pain and minimize postoperative opioid use.^23^ After intercostal nerve cryoanalgesia is applied to T4 to T10 and chest tube placement, the diaphragm is reapproximated using a running No. 1 polypropylene suture. The intercostal space is closed using interrupted No. 2 braided absorbable (polyglactin 910) sutures. The lung is expanded, and the pericostal sutures are secured. A No.1 absorbable monofilament suture (polydioxanone) is used to close the muscular fascia of the chest, first reapproximating the serratus anterior muscle and then the latissimus dorsi muscle, followed by a layer of 2-0 or 3-0 braided absorbable sutures. The skin is closed subcuticularly with 4-0 polydioxanone sutures (or skin staples). The patient is placed in the supine position and the double-lumen tube is exchanged for a single-lumen tube. If there is swelling of vocal cord or coagulopathy requiring abdominal packing, the double lumen endotracheal tube is left in place.

## Postoperative Care

**Figure 6:**
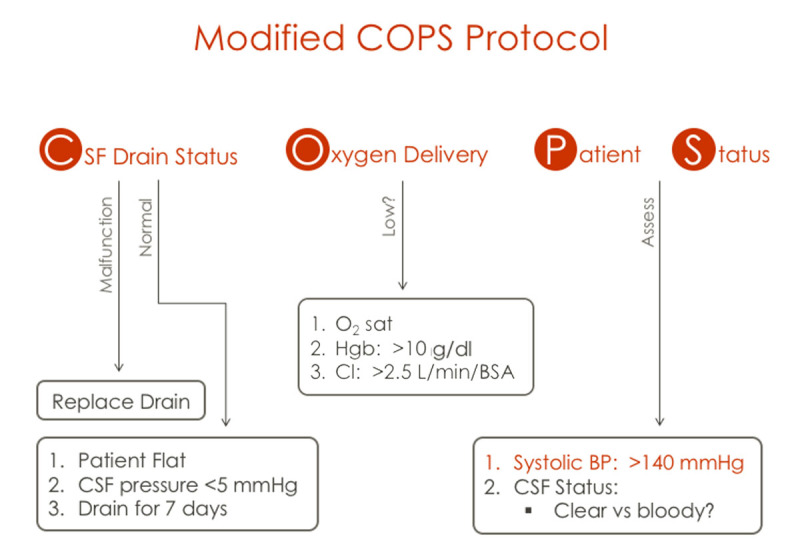
When delayed paraplegia occurs, use a modified COPS protocol: Limitless **C**erebrospinal fluid drainage to maintain pressure below 5 mm Hg; **O**xygen delivery is optimized by keeping hemoglobin above 10 g/dL and cardiac index above 2.5 mL/min/body surface area m^2^; and optimize **P**atient **S**tatus by elevating the systolic blood pressure above 140 mm Hg.

All patients are cared for in an intensive care unit. Postoperative management is also important to avoid delayed paraplegia, and the goal systolic blood pressure should be set above 130 mm Hg by judicious use of blood and blood component therapy.^24^ CSF drainage is continued to maintain CSF pressure below 10 mm Hg. Delayed spinal cord injury (SCI) may occur after a period of normal motor function and remains a devastating complication.^24^ We keep the CSF drain until postoperative day 3 because our data demonstrated the peak incidence is in the second postoperative day.^24^ Once SCI occurs, the CSF drain status, oxygen delivery, and patient status (COPS) protocol (Figure 6) should be instituted: The patient is placed flat and, if the spinal catheter is still in place, then drained freely without maximum amount for 7 days. The hemoglobin level is kept above 10 g/dL, and cardiac output is kept at an optimal level (cardiac index 2.5 L/min/m^2^). In more than 75% of cases, strength may be regained.^25^ MRI or CT imaging should be used to exclude epidural hematoma.

## Summary

The current standard practice of TAAA repair includes: perioperative CSF drainage; left heart bypass with mild hypothermia or cardiopulmonary bypass with moderate/deep hypothermia; sequential clamping; visceral and renal perfusion using roller pump; and aggressive reattachment of patent intercostal arteries within T8 – T12. Our group demonstrated that the use of DAP and CSF drainage, in combination with moderate passive hypothermia, has reduced the overall spinal cord ischemia rate after extent I TAAA from 15% to less than 2%, and after extent II TAAA from 33% (50% with clamp time exceeding 40 minutes in “clamp and go” era) to less than 4%.^26^

## Key Points

- Repair of thoracoabdominal aortic aneurysm requires a coordinated team that includes anesthesia, perfusion, nursing, and surgeon.
- The adjuncts of distal aortic perfusion and cerebrospinal fluid drainage, as well as intercostal artery reattachment, are important for organ protection, including reducing paraplegia.
- Compartmentalizing the procedure, such as sequential clamping and paying attention to details, reduces bleeding and overall complications.
- Although we are in the era of endovascular surgery, open repair remains an important tool for addressing these complex conditions.
